# Sex differences in anxiety and depression: insights from adult rodent models of chronic stress and neural plasticity

**DOI:** 10.3389/fnbeh.2025.1591973

**Published:** 2025-05-14

**Authors:** Rachel Bowman, Maya Frankfurt, Victoria Luine

**Affiliations:** ^1^Department of Psychology, Sacred Heart University, Fairfield, CT, United States; ^2^Hofstra Northwell School of Nursing and Physician Assistant Studies, Hofstra University, Hempstead, NY, United States; ^3^Department of Psychology, Hunter College, New York, NY, United States; ^4^Graduate Center of City University of New York, New York, NY, United States

**Keywords:** chronic stress, sex differences, anxiety, depression, spines, rodents

## Abstract

The often co-morbid conditions of depression and anxiety are the most common mental illnesses and are more prevalent among females than males. Chronic stress paradigms in rodents serve as valuable preclinical models for investigating the factors contributing to these disorders and their neural underpinnings. A variety of chronic stressors are associated with the development of sexually differentiated effects on anxiety- and depressive-like responses in rodents. This review summarizes and discusses common behavioral tasks used to assess anxiety-like (e.g., elevated plus maze, open field) and depressive-like (e.g., sucrose preference, forced swim) behaviors in rodents and discusses evidence of sex differences in these responses. Preclinical chronic stress models also aid in identifying potential mechanisms underlying behavioral changes, including dendritic synaptic alterations in neural circuits affected by stress. Robust sex differences have been observed in stress-responsive brain regions such as the prefrontal cortex, hippocampus, and amygdala. Therefore, applying chronic stress paradigms and assessing their neural effects in rodents may provide crucial insights into the biological basis of sexually differentiated mental illnesses in humans.

## 1 Introduction

The prevalence of mental illness in the USA is higher among females than males and is also more common among the young than the old. For example, in 2022, some 26.4% of females reported some type of mental illness in the past year, compared to 19.7% of males (Results from the 2021 National Survey on Drug Use and Health: https://www.samhsa.gov/data/report/2021-nsduh-detailed-tables, Table 6.7B). Depression and anxiety, often co-morbid, are the most common illnesses (Kessler et al., 2005; Maeng and Milad, 2015). For depression, it is estimated that the incidence is around 5% of the U.S. population, and ~6% of women suffer from depression compared to 4% of men (Results from the 2021 National Survey on Drug Use and Health: https://www.samhsa.gov/data/report/2021-nsduh-detailed-tables, Table 6.7B); smaller samples report a 1.7:1 prevalence of major depressive disorder in women vs. men (Marcus et al., 2005). Similarly, anxiety is higher in females than males. An estimated 19.1% of U.S. adults had any anxiety disorder in 2003 with the prevalence higher for females (23.4%) than for males (14.3%, https://www.nih.gov/health/statistics/any-anxiety-disorder, 2001–2003).

Recent research, both in the U.S. and other countries, supports the view that women are more likely to have internalizing disorders (e.g., anxiety or depression) and men are more like to have externalizing disorders (e.g., antisocial personality or substance use) (Marcus et al., 2005; Ahmed et al., 2018; Bao and Han, 2024; Banos and Miragall, 2024; Eaton et al., 2012). Thus, the need for basic pre-clinical research is paramount, and rodent models play an important role. Yet, it is notable that, until very recently, research using rodents neglected sex as a biological variable and focused almost exclusively on males. However, following the NIH announcement of policies to ensure that funding of preclinical research included both female and male subjects (Clayton and Collins, 2014); more information regarding females has been acquired.

The animal models used to study behavior do not always offer consistent results with respect to mirroring sex differences observed in humans (Borchers et al., 2022). Here, we review the results of studies using chronic stress in adult rodents as a model for understanding the factors promoting mental illnesses and pinpointing the neural bases of anxiety and depression. The validity and utility of this model has recently gained increased credence because studies have documented the stressful effects of the COVID-19 epidemic, including deleterious effects on mental health and other diseases. Previously, it was impossible to assess chronic stress effects in a clinical setting in human populations, and studies focused mainly on acute stress which does not provide relevant information. Ruiz-Villa et al. (2024) studied anxiety symptoms in Colombian health care workers during the COVID-19 pandemic and found that female sex, among others, was associated with higher levels of anxiety. Kuo et al. (2025) investigated elite athletes during the pandemic and found that female athletes reported higher levels of anxiety and depression than male athletes and were more likely to report anxiety, depression and distress. Results from the impact of COVID-19 on college freshmen at the University of Michigan revealed that depression symptoms significantly increased in the pandemic years and became more chronic, especially in females (Turner et al., 2023).

In this review, we consider the effects of chronic stress on adult male and female rodents, but it should also be noted that stress causes neural and behavioral changes across the lifespan- pre-natal, postnatal, pubertal, and at aging (Bowman et al., 2004, 2006; Hodes and Epperson, 2019). In addition, acute stress also affects rodents in a sex-dependent manner (Ludkiewicz et al., 2025) but these research rich topics are outside the scope of this review.

## 2 Behavioral assessments of anxiety and depression in rodents

Some of the most common behavioral models for measuring anxiety- and depressive- like behaviors in rodents are discussed below. A comparison of the strengths and weaknesses of each model is beyond the scope of this review; however, care must be given to the selection of specific tests in relation to other variables and interpretation of results (Snyder et al., 2021; Dalla and Shors, 2009; Rosso et al., 2022) and the basic anthropomorphic assumptions made on the observed animal behaviors (Borchers et al., 2022).

### 2.1 Tests for anxiety-like behavior

Anxiety-like behavioral assessments in rodents include the light dark test, free exploratory paradigm (FEP), the elevated plus maze (EPM), and the open field (OF). These apparatuses place into conflict rodents' natural tendency to explore with their fear of brightly lit spaces (light dark test) (FEP), heights (EPM), and open exposed spaces (OF). Anxiety-like approach-avoidance behaviors in these tasks are also influenced by thigmotaxis, a natural defensive response in which rodents stay close to vertical surfaces, wall-hugging', in an attempt to avoid predators (Lamprea et al., 2008).

The light dark test consists of a compartment divided into a smaller dark “safe” side (1/3 of the box) and a larger illuminated “aversive” side (2/3 of the box). During a typical trial of 5–10 min, the latency to enter, number of entries, and total time spent in the brightly lit side are used as measures of anxiety with longer latencies and decreased total time spent considered to indicate increased anxiety (Rosso et al., 2022; Chen et al., 2024).

The FEP is a box divided into two sides (left and right compartments) separated by a removable barrier, with each compartment further subdivided into three exploratory units interconnected by small openings. A trial consists of the animal subject being allowed to freely explore one compartment side for 24 h followed by a 15 min evaluation period. During the evaluation time, latency to enter the novel side, percent time spent there, and total entries to that side are used as a measure of anxiety (i.e., more entries and time indicating less anxiety) (Oliveira et al., 2014; Teixeira-Silva et al., 2009; Snyder et al., 2021).

The OF is a walled, open-opened arena with the floor marked into grids. The OF provides information regarding locomotor activity (peripheral grid visits or total distance traveled) and anxiety-related behaviors (central grid visits or amount of time spent in center vs. periphery) (Tovote et al., 2015). Fewer inner sector visits are generally considered a measure of increased anxiety (Ennaceur et al., 2006).

The EPM has been referred to as the “gold standard” measure of anxiety-like behaviors in rodents (Biedermann et al., 2017). It is a plus-shaped structure, consisting of two open arms and two enclosed arms, with an open top. The two open arms and the two closed arms are arranged opposite one another around a square center. A typical trial is 5 min during which animal is allowed to freely explore the maze; the number of entries to and time spent in open vs. closed arms is recorded. Avoidance of open arms, as measured by the number of visits to and duration of time spent in open arms is used as an anxiety index, with fewer open arm visits and/or decreased time spent on open arms being indicative of increased anxious behavior (Carobrez and Bertoglio, 2005; Hogg, 1996; Pellow and File, 1986). A further validation that the EPM assesses anxiety is that clinically effective anxiolytic drugs specifically increase and anxiogenic drugs specifically decrease, the number of entries into the open arms, and the time spent there (Braun et al., 2011).

### 2.2 Tests for depressive-like behavior

Measures of depressive-like behaviors in rodents include the learned helplessness model, splash task, novelty suppressed feeding, sucrose preference, and forced swim test. In the learned helplessness model, animals are exposed to an unexpected, unpredictable, or uncontrollable stressor (e.g., shock), and are subsequently tested for deficits in learning an avoidance task (e.g., lever pressing or shuttle box) (Chourbaji et al., 2005; Overmier and Seligman, 1967; Vollmayr and Henn, 2001; Dalla et al., 2010; Dalla and Shors, 2009).

The splash task (Isingrini et al., 2010; Hodes et al., 2015) relies on grooming as a measure of self-care and motivational behavior. Animals are sprayed with a sucrose solution on their backs and time spent grooming during a 5 min trial is recorded. Decreases in grooming are thought to be indicative of depressive-like apathetic behavior (Willner, 2005).

Novelty suppressed feeding uses hyponeophagia, in which exposure to a novel environment suppresses feeding behavior, to assess depressive-like symptoms in rodents (Hodes et al., 2015; Belovicova et al., 2017; Santarelli et al., 2003). Animals are food restricted overnight and then placed in a novel environment with access to a food pellet. The latency to eat is measured (with a typical 10 min maximum) and longer times are considered characteristic of increased depressive-like symptoms.

The most common assessments of depression-like behaviors in rodents are the sucrose preference test (SPT) and forced swim test (FST). The SPT uses a two-bottle choice paradigm where rodents can freely choose between drinking regular water or sucrose (or saccharin) water. A core attribute of depression is anhedonia, the loss of experiencing reward or pleasure. In the SPT, the amount or percentage of sucrose consumption is measured. Decreases in sucrose preference indicate anhedonia and is interpreted as a behavioral measure of depression in rodents (Primo et al., 2023; Liu et al., 2018).

The FST is based on rodents' natural tendency to escape water and measures behavioral despair in rodents through a two-trial forced swim session in a cylinder container (Porsolt et al., 1978; Kraeuter et al., 2019; Dalla et al., 2010). The day one trial is typically a 10- or 15-min session followed the next day by a 5 min test session. Behaviors recorded include time spent swimming, escape or climbing behaviors, and time spent immobile or “floating.” Animals spending less time actively swimming and/or more time immobile are exhibiting greater depressive-like behaviors. However, it should be noted that some researchers indicate that immobility may not reflect depression but is instead active coping (Molendijk and De Kloet, 2015). This criticism highlights that a variety of tests should be applied to assess anxiety and depression in rodents.

Another important consideration is that the characteristics of the described testing apparatuses (e.g., dimensions and materials) and specific procedural methodologies used vary considerably across laboratories which impact behavioral measurements (Gaspar et al., 2023; Snyder et al., 2021). For example, the open field test can be circular or round (Seibenhenerr and Wooten, 2015), trials are of different durations including 6 (Beck and Luine, 2002; Bowman and Kelly, 2012), 10 (Seibenhenerr and Wooten, 2015), and 15 min (Borchers et al., 2022), and different behaviors are measured beyond locomotion including wall climbs, rears, and fecal boli (Seibenhenerr and Wooten, 2015). A further complication is that behavioral measures within a task (e.g., anxiety as measured on the open field using locomotor activity and defecations) are not always correlated (Ramos et al., 2008), and measures of anxiety- or depressive-like behaviors are not always correlated across tasks (Snyder et al., 2021; Rosso et al., 2022). These issues highlight that careful consideration must be taken when interpreting phenotypical anxiety- and depressive-like behavioral outcomes.

## 3 Baseline sex differences in measures of anxiety and depressive-like behaviors

In assessing effects on anxiety and depressive-like behaviors, it is important to note that there is a strong body of evidence that anxiety-like behaviors in rodents on the EPM and OF are sexually differentiated (Beck and Luine, 2002; Bowman et al., 2009; Luine et al., 2007; Knight et al., 2021; Scholl et al., 2019; Imhof et al., 1993; Domonkos et al., 2017; Johnston and File, 1991; Ramos et al., 2002). However, others report no sex differences (Albrechet-Souza et al., 2020; Mansouri et al., 2019; Yang et al., 2019) but these seemingly conflicting results have been attributed to small sample size and apparatus characteristics such as EPM arm width (Gaspar et al., 2023; Knight et al., 2021).

On the EPM, females showed less anxiety-like behaviors than males by spending more time in the open arms and making more entries to the open arms (Knight et al., 2021; Scholl et al., 2019; Borchers et al., 2022). Importantly, this sex difference was not impacted by estrous cycle stage (Scholl et al., 2019), a finding echoed by meta-analyses showing no differences in male and female data variability on behavioral measures of anxiety (Kaluve et al., 2022; Beery, 2018; Becker and Koob, 2016).

Similar findings have been observed on the OF. On a “free open field” in which animals can enter the field on their own, rather than starting the trial by being placed in the field, young adult control female rats showed less anxiety-like behaviors than young control males as measured by the latency to enter open field (Beck and Luine, 2002). Other OF behavioral measures also show sex differences. Females made more OF inner sector visits than males (Bowman et al., 2015; Knight et al., 2021; Pavlova et al., 2020) and spent more time in the inner sectors than males (Borchers et al., 2022; Burke et al., 2016).

The finding that females show less anxiety-like behaviors than males may appear inconsistent with the general behavioral phenotype for humans, in which females are more prone to anxiety disorders than males (Bangasser and Valentino, 2014). One possible explanation for this inconsistency is that the sex differences in rodents described above occur under controlled conditions (e.g., no exposure to stress prior to behavioral testing) whereas in humans, stressful experiences are a common factor in the etiology of anxiety disorders (Bangasser and Valentino, 2014; Brady and Sinha, 2005) as is alcohol and drug consumption which can alter anxious behaviors (Luine et al., 2017a). Additionally, the tasks described above were originally developed for use in male rodents and were later applied to female subjects with little consideration given to the fact that basic behavioral sex differences may mask or alter the typical anxiety- or depressive- like behavioral outcomes being measured. For example, in the EPM the majority of behavioral variance in males can be attributed to anxiety but in females the majority of variance can be attributed to locomotor activity (Fernandes et al., 1999).

Most behavioral assessments are conducted in animals exposed to stress or drug exposure resulting in limited data on the existence of baseline sex differences in depressive-like behaviors. However, it has been observed that female rats consumed more sucrose solution than males on the SPT (Grimm et al., 2022; Dalla et al., 2005), yet others have reported no sex differences in sucrose preference (Tordoff et al., 2008). On the FST, female rats show longer immobility times than do males indicating a sex difference in behavioral despair (females greater than males) (Kokras et al., 2015; Drossopoulou et al., 2004; Pitychoutis et al., 2009; Dalla et al., 2008). Taken together, it appears that females may show more depressive-like behaviors on the FST in a way that is consistent with vulnerability to depression seen in human females.

Thus, it appears that rodent subjects may be starting at different levels of both anxiety- and depressive-like states before experimental manipulations are instituted. These innate, baseline sex differences add another level of complexity which masks interpretation of experimental effects. In addition, floor or ceiling effects might be encountered following experimental manipulations which may further complicate conclusions.

## 4 Chronic stress effects on measures of anxiety

As shown in Table 1, measures of anxiety-like behaviors in rodents have frequently been studied following chronic stressors such as daily restraint (typically 6 h/day) or chronic unpredictable stress (CUS) exposure, and there is evidence that stress alters baseline anxiety sex differences. Many studies have utilized only male subjects and have shown that both restraint stress, of varying durations (Moreno-Martinez et al., 2022; Nagaoka et al., 2016; Chiba et al., 2012), and CUS (Adavi et al., 2024) increases anxiety in males. Anxiety, as measured on the EPM and OF, are increased in both male and female rodents following chronic stress (Luine, 2016), although these effects depend on the stress duration. Our laboratories have shown that 7 days of restraint stress increases EPM anxiety measures in males but not females (Bowman et al., 2009; Gomez et al., 2013; Luine, 2014), however, females also show increased anxiety on the open field following longer restraint durations of 21 (Beck and Luine, 2002; Bisagno et al., 2004; Huynh et al., 2011), and 35 days (Bowman and Kelly, 2012).

**Table 1:** Effects of chronic stress on anxiety-like behaviors in adult rodents.

**Duration/Subject**	**Stressor**	**Task**	**Males**	**Females**	**References**
6 days Mouse	SCVS	EPM	=	=	Hodes et al., 2015
6 days Mouse	CUS	EPM	=	=	LaPlant et al., 2009
7 days Rat	Restraint	EPM	↑	=	Bowman et al., 2009
7 days Rat	Restraint	EPM	↑	=	Gomez et al., 2013; Gomez and Luine, 2014
10 days Rat	Social defeat	EPM	↑	Not tested	Stickling and Rosenkranz, 2024
15 days Rats	CUS	EPM OF, center visits	↑	↑	Xia et al., 2023
21 days Rat	Restraint	OF, entry latency	Not tested	↑	Bisagno et al., 2004
21 days Rat	Restraint	OF, center visits	↑	↑	Beck and Luine, 2002
21 days Rat	Restraint	OF, center visits Dark phase	↑	=	Huynh et al., 2011
21 days Rat	Restraint	EPM, dark phase	=	↑	Huynh et al., 2011
21 days Rat	Restraint	OF, center visits Light phase	=	=	Huynh et al., 2011
21 days Rat	Restraint	EPM, light phase	=	=	Huynh et al., 2011
21 days Rat	Intermittent restraint	OF, anxiety index	=	=	Peay et al., 2020
35 days Rat	Restraint	OF, center visits	Not tested	↑	Bowman and Kelly, 2012
42 days Rat	Intermittent restraint	OF, anxiety index	=	↓	Peay et al., 2020
50 days Rat	Restraint^*^	EPM	↑	=	Noschang et al., 2009
84 days Rat	Isolation	OF, center visits	↑	Not tested	Lucindo et al., 2025

Adult rats received chronic stress. Restraint is 6 h/day for number of days shown except ^*^ where indicates restraint was for 1 h/day for 5 out of 7 days.

CUS, Chronic Unpredictable Stress; SCVS, Subchronic Variable Stress.

Anxiety was measured on the elevated plus maze (EPM) and open field (OF) where upward arrows indicate an increase in anxiety measure and = indicates no change. Adapted from Luine et al. (2017b).

Table 1 shows studies in which anxiety was measured following a variety of chronic stress paradigms in male and female rodents (with a few exceptions in which only one sex was examined). Stress-induced increases in anxious behaviors are observed in males sooner (e.g., 7 days restraint) than females; thus, female rats appear more resilient than males to stress effects on anxiety-like behavioral measures. Interestingly, while not the focus of this review, the same temporal pattern of differential stress duration effects in males and females is observed in cognition. For example, 13 days of CRS enhances spatial memory in males, but longer periods of 21 days cause impairments; however, females have enhanced spatial memory following 21 days of CRS and no behavioral change following 28 or 35 days (for review see Luine et al., 2017a; Bowman et al., 2022). These temporal differences in stress effects on anxiety behaviors may be an important consideration when designing experiments.

## 5 Chronic stress effects on depressive-like behavior

A variety of stressors and behavioral tests have been applied to investigate the effects of chronic stress on depressive-like behaviors in rodents, described above. As with anxiety, depression appears to be a consequence of repeated, chronic stresses, and both sexes appear vulnerable. Table 2 lists studies that utilized both sexes (with a few exceptions) and indicates that females appear affected after shorter stress regimens than males. This pattern is opposite to stress effects on anxiety where males appear affected after fewer days of stress than females. For example, following 6 days of subchronic variable stress (SCVS), female mice exhibited depressive-like symptoms using the splash test, novelty suppressed feeding, sucrose preference, and the forced swim task (FST) while males did not (Hodes et al., 2015; LaPlant et al., 2009). However, following 7 days of chronic restraint, neither male nor female rats were affected in the FST (Gomez et al., 2012; Luine et al., 2017a), but both rat sexes showed anhedonia at 7, 14 and 21 days on the sucrose preference test (Luine et al., 2017a), and a variety depressive like behaviors were exhibited by both sexes at 21 through 28 days of stress (see Table 2). Notable exceptions to this pattern occurred when rats were tested in the dark as compared to light phase (Huynh et al., 2011). While there have been more recent studies than those reported in Table 2, and the importance of sex as a biological variable has been advocated, surprisingly, recent experiments did not include both sexes and are therefore not discussed here (see Stickling and Rosenkranz, 2024; Conrad et al., 2024 for example).

**Table 2:** Effects of chronic stress on depressive-like behaviors in adult rodents.

**Duration/Subject**	**Stressor**	**Task**	**Males**	**Females**	**References**
6 days Mouse	SCVS	Splash	=	↑	Hodes et al., 2015
6 days Mouse	SCVS	NS feeding	=	↑	Hodes et al., 2015
6 days Mouse	SCVS	SP	=	↑	Hodes et al., 2015
6 days Mouse	CUS	FST	=	↑	LaPlant et al., 2009
7 days Rat	Restraint	FST	=	=	Gomez et al., 2013; Gomez and Luine, 2014
15 days Rats	CUS	FST SP	↑	↑	Xia et al., 2023
7–21 days Rat	Restraint	SP	↑	↑	Luine et al., 2017a
21 days Rat	Restraint	FST SP Dark phase	=	=	Huynh et al., 2011
21 days Rat	Restraint	FST SP Light phase	↑	↓	Huynh et al., 2011
7–28 days Rat	SCVS	SP	↑	↑	Dalla et al., 2010
7–21 days Rat	Restraint	SP	↑	↑	Luine et al., 2017a
8–26 days Rat	CMS	SP	↑	Not tested	Gronli et al., 2005
84 days Rat	Isolation	SP	↑	Not tested	Lucindo et al., 2025

Adult rats received chronic stress. Restraint is 6 h/day for number of days shown.

SCVS, subchronic variable stress; CMS, chronic mild stress; CUS, chronic unpredictable stress; SP, sucrose preference; FST, forced swim task; NS feeding, novelty suppressed feeding. Upward arrows indicate an increase in depression measure, downward arrows a decrease, and = indicates no change.

There are a number of caveats which must be considered regarding the pattern of stress effects on depressive-like behaviors in the aforementioned studies. First, shorter chronic stress regimens have only been completed in mice vs. rats; therefore, it remains possible that mice and rats respond differently to stress. Thus, whether female rodents are more susceptible than male rodents to the depressive effects of chronic stress requires further testing. In addition, a greater variety of tests need to be applied to both sexes after longer periods of stress since mainly sucrose preference tests have been used. However, a positive take-away from Table 2 is that a variety of stressors, when applied chronically, are associated with depressive responses in rodents. Thus, this paradigm appears to be a valid pre-clinical paradigm for investigating the sources and basis for human depression.

## 6 Potential neural plasticity mechanisms underlying sex differences in anxiety and depression

There is increasing evidence that anxiety and depression involve neural plasticity (Iqbal and Ma, 2020; Qiao et al., 2016; Leuner and Shors, 2013; Iqbal et al., 2024). Given the difficulty in assessing brain alterations in humans, it is necessary to use animal models. In animals, the aforementioned chronic stress models have been used to assess the potential role of neural plasticity in anxiety and depressive like behaviors.

**Figure 1:**
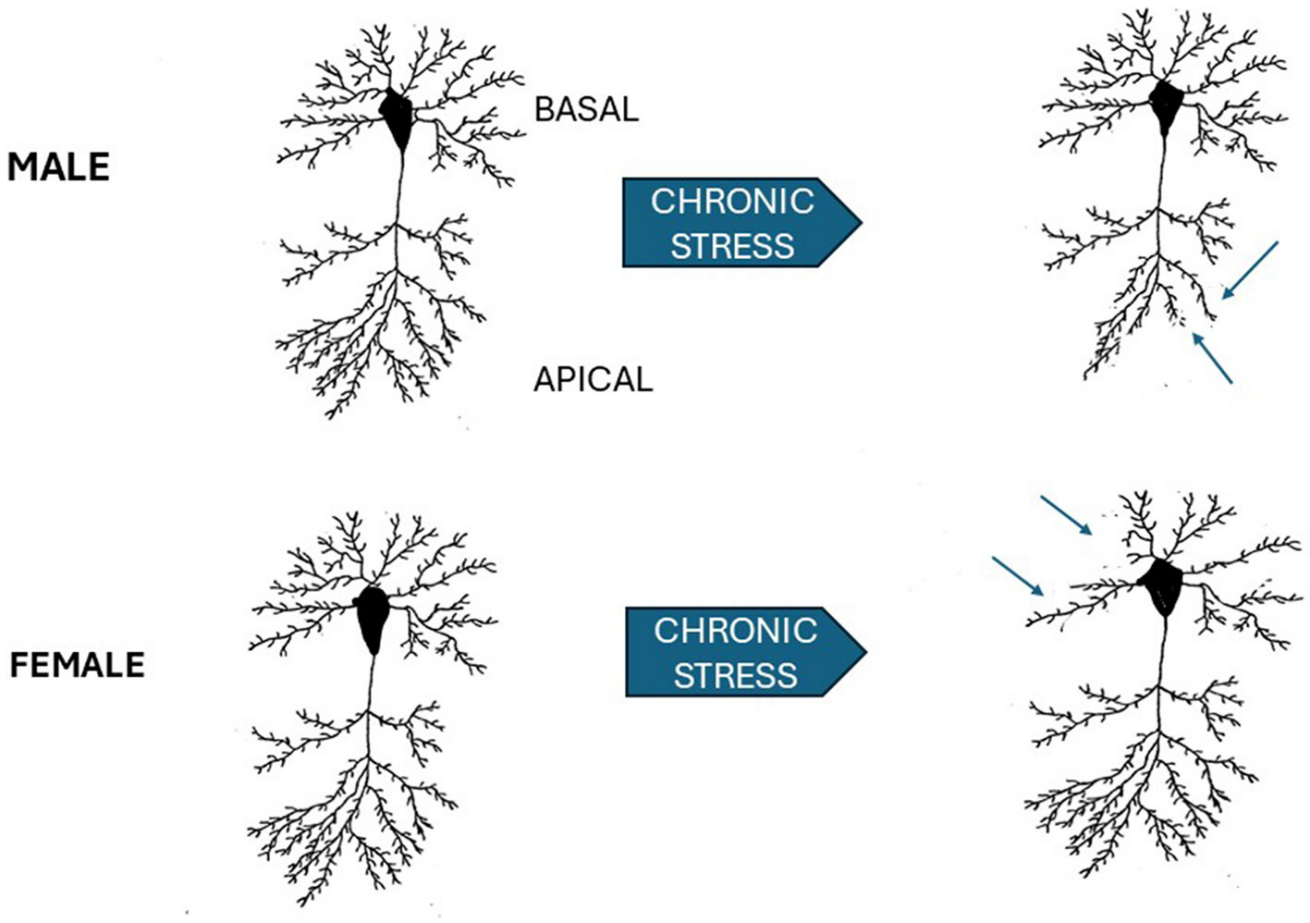
Schematic of sex differences in chronic stress effects on hippocampal CA3 pyramidal neurons. Neurons in the CA3 subfield of male, upper left, and female (lower left) are shown prior to 3 weeks of 6 h/day of restraint stress and following stress. Arrows point to the apical dendrites in the male which show dendritic retraction. In the female (lower left), apical dendrites are not affected, but basal dendrites are retracted following stress. Male results adapted from Watanabe et al. (1992). Female results adapted from Galea et al. (1997).

The brain regions involved in mediating stress induced responses include the hippocampus, prefrontal cortex, and the amygdala which are extensively interconnected and alterations in neural structure have been demonstrated in these regions following chronic stress (Laine and Shansky, 2022). In male rats, chronic restraint stress causes atrophy of apical dendrites in pyramidal cells in CA3 of the hippocampus but does not alter basal dendrites in the same cells (Magarinos and McEwen, 1995; Watanabe et al., 1992; see Figure 1). Chronic stress effects have also been reported for hippocampal CA1 pyramidal neurons but they are more variable and, in general, appear more resistant to stress (Shansky and Morrison, 2009; Cook and Wellman, 2004; Moench and Wellman, 2015; Qiao et al., 2016). In the male mouse basolateral amygdala, chronic restraint stress induces neuronal hypertrophy, and increased spine density in a circuit specific manner. Projection neurons from the basolateral amygdala to the ventral hippocampus that are known to mediate anxiety showed an increase in dendritic spine density following stress (Zhang et al., 2019). In contrast, in the central nucleus of the amygdala, a decrease in dendritic spine density is seen after chronic restraint stress (Moreno-Martinez et al., 2022).

As with most studies, there is less data in female animals. However, 21 days of chronic restraint stress induces different effects in the sexes. There is retraction of apical dendrites in the CA3 region of the hippocampus in male, but not female, rats (Galea et al., 1997). In female rats retraction is seen in basal dendrites (see Figure 1). A sex difference in stress effects in CA1 in the ventral hippocampus has also been reported (Rico et al., 2015). Stressed female rats show a decrease in basal dendritic spines, an increase in the number of apical intersections while there were no stress effects in male rats.

Sex differences in the response to stress have also been found in the prefrontal cortex. As indicated above, apical dendrites and dendritic spine density are decreased in male rats after chronic stress, while females either exhibit no dendritic changes or may even exhibit dendritic hypertrophy depending on stress duration (Garrett and Wellman, 2009; Moench and Wellman, 2017; Shansky et al., 2010). In addition, these studies showed a sex difference in dendritic arborizations in these neurons with males having greater arborization than females. In a study in which mice were subjected to different stressors 1 h daily for 6 days, only OVX female mice were found to be susceptible to the stress (Iqbal and Ma, 2020). The OVX female mice had significantly higher levels of corticosterone, increased spine density on prefrontal cortex pyramidal neurons, increased immobility time for several behavioral tests and decreased sucrose consumption, which is consistent with anhedonia, in comparison to intact males and sham operated females. These studies suggest that neural networks related to depression are differentially affected during stress and this may help explain sex differences in the incidence of depression. This conclusion is supported by the data from Bittar et al. (2021) who compared projections from the prefrontal cortex to the ventral tegmental area (VTA) or the nucleus accumbens following chronic variable stress in mice. Although both males and females exhibited depressive- like behavior, there were more drastic reductions in dendritic complexity in neurons projecting to the VTA in males than in females.

When examining potential sex differences in anxiety and depression, the role of estrogen in altering stress responses may be important. Low estrogen levels are consistent with increased anxiety and depression and estrogen replacement has been shown to improve anxiety in both rodents and humans (Iqbal et al., 2024; Puga-Olguin et al., 2019). In addition, in aged rats when estradiol levels plummet, females are more anxious than males, a reversal from the pattern in young adults (Bowman et al., 2006). Moreover, it has been well documented by us and others that dendritic spine density in the hippocampus and prefrontal cortex fluctuates with estrogen levels. When estrogen levels are high spine density is increased on pyramidal neurons (Frankfurt and Luine, 2015; Luine and Frankfurt, 2012, 2020; Gould et al., 1990; Woolley et al., 1990), and the inverse has also been shown (Wallace et al., 2006, 2007; Gould et al., 1990; Woolley et al., 1990). A possible interaction between estrogens and stress was shown by McLaughlin et al. (2005) who found that chronic restraint stress in ovariectomized female Wistar rats caused CA3 apical dendritic retraction, similar to stress effects in males (Watanabe et al., 1992), and different from effects in gonadally intact females (Galea et al., 1997). Moreover, stress in ovariectomized rats increased the proportion of CA1 spine heads compared with controls. Clearly these results suggest that the presence of estrogen could be responsible for potential sex differences in neural networks, and this might explain the sex differences observed in both pre-clinical and clinical conditions.

## 7 Conclusions

Results reviewed here and tabulated in Tables 1, 2 show that a variety of stressors, when applied chronically, are associated with development of sexually differentiated effects on anxiety and depressive-like responses in rodents. Thus, chronic stress paradigms appear to be valid pre-clinical paradigms for investigating the sources and neural basis for these sexually differentiated human illnesses. Importantly, several different kinds of stressors, such as restraint, social isolation, intermittent stress, are potent, but it must be noted that the time course of expression and the sensitivity of either sex to specific stressors maybe different. Thus, care must be exercised in application of stress paradigms, and effects on anxiety and depression must be documented.

Preclinical chronic stress models also appear useful in determining possible underlying mechanisms contributing to behavioral changes. The possible role of dendritic and synaptic alterations in neural systems subserving stress effects were discussed here. Robust sex differences in response to stress are found in brain systems subserving these behaviors, prefrontal cortex, hippocampus, and amygdala. Pyramidal neurons of the hippocampus show dendritic retractions in apical CA3 neurons of males whereas females show retractions in basal CA3 neurons following chronic stress. In the medial prefrontal cortex, chronic stress is associated with retraction of neurons in male rats whereas females either exhibit no dendritic changes or may even exhibit dendritic hypertrophy. Stress effects on dendritic spine density are also found, but further investigations of possible sex differences are necessary. Thus, the application of stress and assessments of neural effects in rodents may provide seminal information for unraveling the bases of some human mental disorders.
